# If you come from a well-known organisation, I will trust you: Exploring and understanding the community’s attitudes towards healthcare research in Cambodia

**DOI:** 10.1371/journal.pone.0195251

**Published:** 2018-04-18

**Authors:** Sreymom Pol, Shivani Fox-Lewis, Leakhena Neou, Michael Parker, Patricia Kingori, Claudia Turner

**Affiliations:** 1 Cambodia Oxford Medical Research Unit, Siem Reap, Cambodia; 2 Faculty of Tropical Medicine, Mahidol University, Bangkok, Thailand; 3 Centre for Tropical Medicine and Global Health, Nuffield Department of Clinical Medicine, University of Oxford, Oxford, United Kingdom; 4 Angkor Hospital for Children, Siem Reap, Cambodia; 5 The Ethox Centre, Nuffield Department of Population Health, University of Oxford, Oxford, United Kingdom; TNO, NETHERLANDS

## Abstract

**Objective:**

To explore Cambodian community members’ understanding of and attitudes towards healthcare research.

**Design:**

This qualitative study generated data from semi-structured interviews and focus group discussions. This study was conducted at a non-governmental paediatric hospital and in nearby villages in Siem Reap province, Cambodia. A total of ten semi-structured interviews and four focus group discussions were conducted, involving 27 participants. Iterative data collection and analysis were performed concurrently. Data were analysed by thematic content analysis and the coding structure was developed using relevant literature.

**Results:**

Participants did not have a clear understanding of what activities related to research compared with those for routine healthcare. Key attitudes towards research were responsibility and trust: personal (trust of the researcher directly) and institutional (trust of the institution as a whole). Villagers believe the village headman holds responsibility for community activities, while the village headman believes that this responsibility should be shared across all levels of the government system.

**Conclusions:**

It is essential for researchers to understand the structure and relationship within the community they wish to work with in order to develop trust among community participants. This aids effective communication and understanding among all parties, enabling high quality ethical research to be conducted.

## Introduction

One of the main principles of community-based research is to ensure that potential participants are provided with enough information to make sure that they are informed of the goals of research and understand its aims [1]. It is also increasingly argued that community-based research should involve prioritising community members’ understanding of research in general as it helps to promote better communication, transparency and is respectful to the community [2, 3].

In practice, most studies of community understanding of healthcare research are undertaken after the research has been conducted and generally focus on the individual participants of that research. There is little literature on community-wide (including a range of stakeholders) understanding of research prior to individual participation [3]. As a consequence, most recommendations on community understanding of healthcare research by researchers and institutions are based on a narrow set of assumptions and views from the accounts of individuals who have participated in their research, which may not be representative of the community as a whole [2, 4].

Given the increasing importance and volume of community-based research activities in low income settings, empirical research on community understanding of research in such contexts prior to individual participation is urgently needed [5].

### Healthcare research in Cambodia

While the amount of research conducted in Cambodia is increasing, generally, knowledge about research is very limited [5]. Previous work has demonstrated that most Cambodians have not encountered concepts such as ‘research ethics’ or experienced giving their consent for research [6, 7]. Related studies suggested that the lack of prior exposure to these concepts could be an explanation for the reported high rates of refusal and loss to follow up for healthcare research conducted outside of hospital settings [6, 8]. In addition, social, political, historical and economic issues have been presented as possible explanations for refusal [8]. This suggests that exploring community members’ understanding of terms and concepts such as “healthcare research” and “consent” could assist in the development of more locally relevant forms of communication, informed by a wide-range of stakeholders and improve transparency in healthcare research in Cambodia [2].

For example, studies have shown that community-wide beliefs around the method for obtaining consent can impact on the decision to participate [9]. In a study conducted in Sotnikum health district, Cambodia, participants were eager to be involved in healthcare research until they were asked to sign consent forms or give their thumbprints [7], the historical and political climate being an important factor in them providing a thumbprint rather than a verbal agreement or signature. Similarly a Ugandan study found that potential participants became very concerned that they could lose their property if they provide a thumbprint or signature as this method of consent resonated with historical practices which were used to surreptitiously remove land rights from unsuspecting members of the population [6]. The difference in interpretation of the practice of providing a signature between researchers and community study participants demonstrates how a common practice which might be relatively unproblematic in some settings may not be aligned with participants’ beliefs about how best to gain their consent. This provides one example of why research investigating community members’ understanding of research processes and how they should be adapted to concur with community values is of great importance.

This study explored Cambodian community members’ understanding of healthcare research and their attitudes towards research practices, with a specific focus on methods for obtaining consent. By gaining an understanding of such factors, researchers can ensure their practice is acceptable to the community.

## Method

### Design

This qualitative study involved semi-structured interviews (SSIs) and focus group discussions (FGDs) with community members living in Siem Reap province, Cambodia.

**Fig 1:**
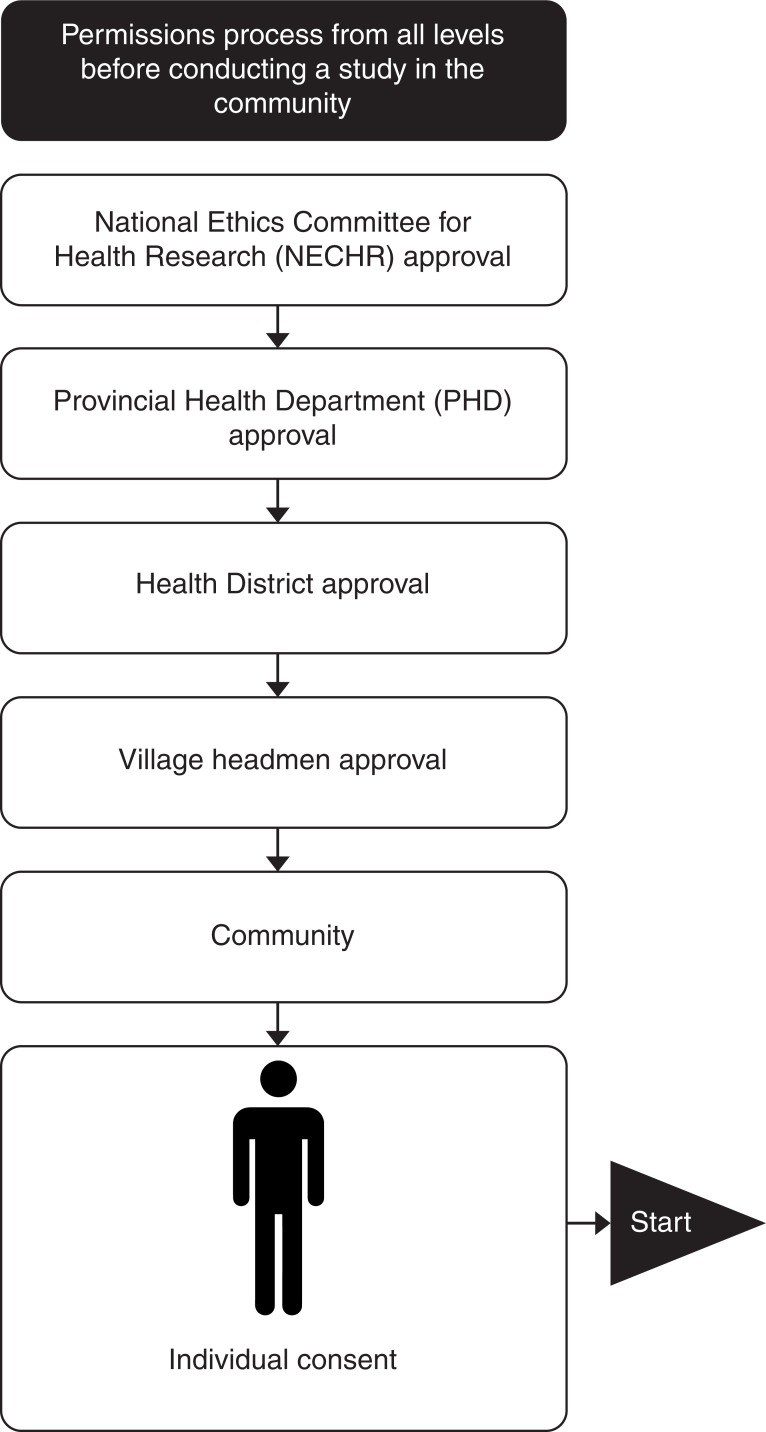
Permission process from all levels before conducting study in the community.

Prior to data collection, due processes of obtaining approval were followed, requiring approval from authorities at each level of the organisational structure (Fig 1).

### Setting

The study was conducted in two locations Angkor Hospital for Children (AHC) and villages in Sotnikum health district [10]. Sotnikum health district comprises rural and peri-urban villages. AHC is a not-for-profit paediatric referral hospital with 91 beds and offers outpatient, inpatient and intensive care to all children who present to the hospital (not limited to Siem Reap province).

### Recruitment of participants

Purposive sampling at AHC was conducted by visiting each inpatient ward to recruit parents and grandparents of children admitted to the hospital. Any such parent or grandparent that was present that day was eligible for participation. Data collection and analysis occurred simultaneously in an iterative manner, and as such participants were recruited until data saturation was reached.

At Sotnikum health district, sampling involved visiting one village at a time and inviting eligible villagers (aged 18 or above) present that day to participate. Village headmen were recruited first. A village headman is the administrative and societal leader of that village. Three village headmen were recruited following which data saturation was reached. After gaining permission from the village headmen (Fig 1), villagers were recruited. Again, data collection and analysis occurred concurrently, and villager recruitment was conducted until data saturation was reached.

In total, 27 participants were enrolled in this study. Ten participants were involved in SSIs (five from AHC and five from the Sotnikum villages) and 17 were involved in the four FGDs (nine from AHC and eight from Sotnikum villages). Socioeconomic characteristics are shown in [S1 Table].

### Data collection

Data collection occurred from February to June 2016. SSIs and FGDs were conducted until data saturation was reached. In total, there were ten SSIs and four FGDs. The interviews and discussions were conducted in Khmer (national language) by Khmer-speaking members of the study team. The topic guide included asking whether participants had heard of “research” before and what their understanding of the term was, their opinions on research and what makes research good, and what researchers should prioritise when conducting community-based research. Specifically, participants were asked which methods of consent they prefer and why.

It is worth noting that there was often a difference in understanding of the term “research” between participants and researchers, with many participants confusing research with healthcare activities. This was an important finding of this study (discussed in the results section) but one which also shaped its conduct. During the process of obtaining the consent of potential participants examples of healthcare research activities were used to explain what research is, to enable participants to understand and answer the questions which were part of this study.

In this study, participants were given study information sheets in Khmer prior to the SSIs and FGDs. In the case of potential participants who were illiterate, the study was explained verbally. Participants had the opportunity to have all their questions answered before they were enrolled into the study. Prior to the SSIs and FGDs, participants were asked a range of socioeconomic questions to gather background demographic information. A key feature of this study was that participants were given a choice of how to consent to their participation through either verbal, signature, or thumbprint approval [11].

### Data management and analysis

Data analysis was conducted iteratively, with the topic guide amended as necessary to pursue pertinent questions and emerging themes. The recordings were transcribed into a Microsoft Word document and translated into English. The translated transcripts were exported into a qualitative data management software package (NVivo; QSR International Pty Ltd. Version 11, 2015). Thematic content analysis was used, deriving themes from the data empirically. No prior assumptions were made about the data, allowing an inductive approach to analysis. Emerging themes were discussed and agreed upon by the study team [12, 13]. The key themes were discussed and agreed upon by the study team.

### Ethical review

Ethical approval was obtained from Angkor Hospital for Children, Institutional Review Board (AHC IRB: 0101/16), National Ethics Committee (NEC: 052 NECHR), and the University of Oxford Tropical Research Ethics Committee (OxTREC: 581–16).

## Results

Our findings report on three main areas: understanding of research, attitudes towards research and attitudes towards consent. Within “understanding of research” two themes emerged: Definition of research, good and bad research practices, “attitudes towards research” two themes emerged: trust, responsibility, and under “attitudes towards consent” two themes emerged: methods of obtaining consent and the need for incentives. In general, responses from participants recruited from the hospital did not differ greatly from responses from participants recruited from the villages.

**Fig 2:**
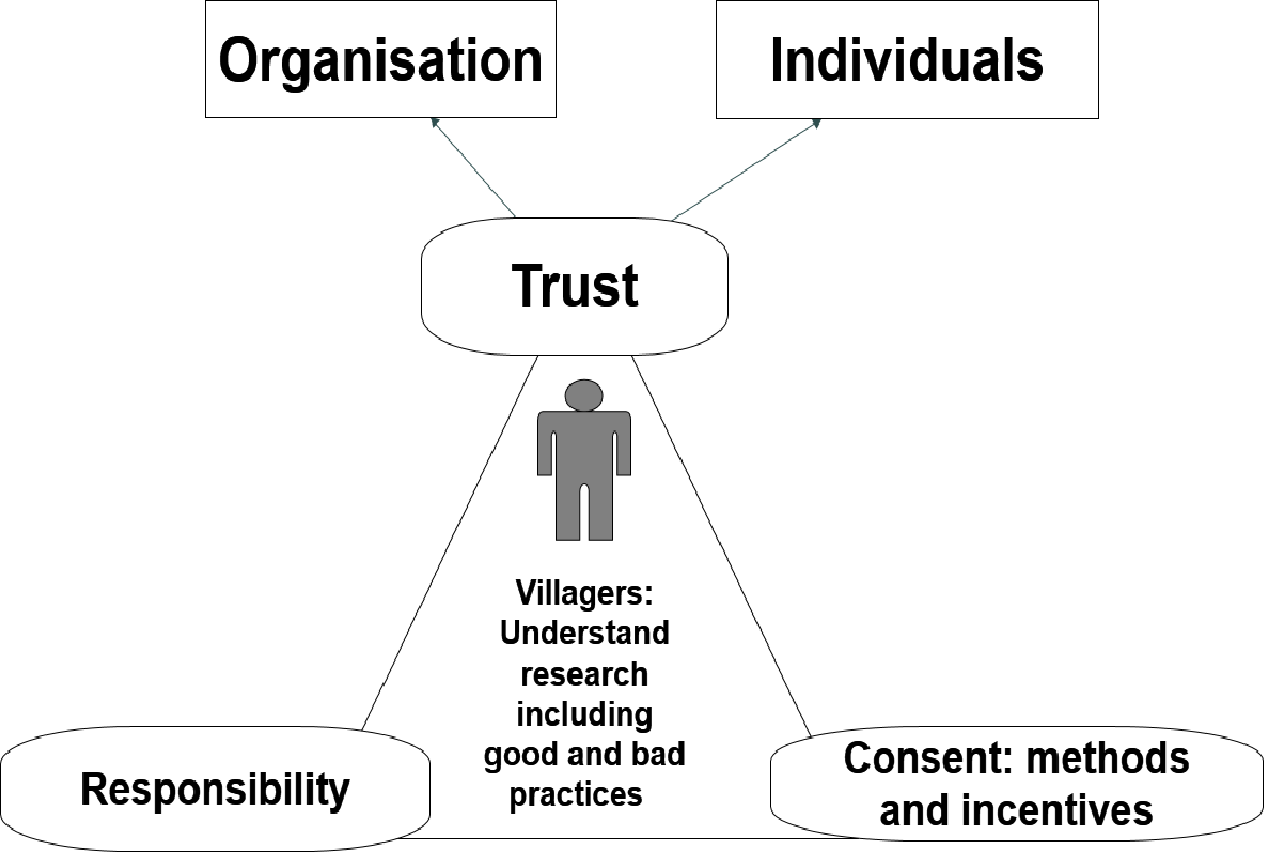
Summary of the interlinked themes for healthcare research.

An important finding of this study was that all the themes discussed are interlinked (Fig 2).

### Understanding of research

#### Definition of research

In Khmer, the term "research" is usually translated as “to look for", “to find”, and “to search for”. As a consequence, when participants were asked about “research”, they believed this referred to looking for people who are poor or sick and who live in rural areas. They commonly thought that research is about directly helping people because many of those perceived to be conducting “research” are non-governmental organisations (NGOs):

Yes, I heard about research before in the villages. It is like there are organisations come and search for poor people. My family was selected. They come to my house take the picture of my family standing in front of my cottage, after that I got the support such as rice, and some cloth and money and the poverty card.

(Mother, SSIs, Hospital)

The above quote illustrates the fact that community members did not always have a clear understanding of the word “research”. In this study, villagers were asked if they had experienced or seen anyone conducting any health-related activities in their community. To explain the word “research” examples were given e.g. they were told that “a team wants to know how much malaria there is in the village and they will be coming to the village with questionnaires and want to take blood samples to test for malaria to see how many people have it”. This example was given as a prompt to explore their understanding and associations with the term research, without depending upon prior knowledge of the term.

This exercise illustrated that untangling research from other health-related activities was difficult for most of those questioned. Consequently many villagers perceived both general health-related interventions and healthcare research as different forms of healthcare practice which were conducted if they were sick.

[General] vaccination is called research…I have heard about research as looking for disease, but I never saw anyone doing it in my village

(Village Headman, Villages)

Another participant mentioned an episode where people came to draw blood in his village, to check for malaria and he agreed to take part only because he believed himself to be sick and was concerned that he might have malaria. This participants adds that:

Well, first there was an announcement about them (name of NGO) who came to treat malaria. And they came to do the blood test to check if we are sick or not.

(Father, SSIs, Hospital)

#### Good and bad research practices

The taking of blood was seen as significant by participants and as creating certain important expectations. Many participants mentioned that if their blood was taken as part healthcare research then they would want to know the result of that research. Even if individual results could not be given, they would still expect general results as part of their idea of good research. The lack of these results was reported to make participants feel like their given sample, and their time, were wasted and they were left in ignorance. This parent argues that:

Well, logically, if they draw blood, we should get the result, no matter how long it takes. But if we don’t get results at all, wouldn’t it imply that the blood was not tested and just drawn and left?

(Parent, SSIs, Hospital)

If the research involved gaining blood samples then those involved in this study said if they felt sick, they would allow researchers to take blood in the hope of gaining results. For healthy participants, they would consent to blood withdrawal because they would want information on any unknown illness. If the blood test was for children or for their future benefit, most parents would give their consent if the volume of blood taken was small.

Participants were also asked if they have any reservations about their samples being taken abroad to be studied. A common response was that they did not have any concerns. Most mentioned that when a blood sample was given, they no longer considered the sample to be “part of them”, therefore, it was then up to the researchers what they did with it as would not harm them. This participant’s views were typical of many asked, in stating that:

Honestly, it is really up to you (researcher). You can take the sample to do the test anywhere you want because after you take it, the sample becomes yours.

(Father, SSIs, Hospital)

The institutional and personal trust implicit in this quote will already have been established in order for the participant to give their blood sample. Therefore, having given the sample, they would trust the researcher and the organisation with regard to their use of it.

Moreover, participants said that understanding the reasons for and outcome of research was important to them. Participants explained that research should involve ‘helping people’ in the longer term even if it is not beneficial to the participants immediately and feeding findings and results back to the community. One participant gave their opinion as follows:

To me, doing research about health by drawing blood to search for new discoveries is the best. They are helping a lot of people by looking for our common health problems and also can help our kids in the future. It should be done in this village.

(Villager, SSIs, Villages)

This participant associated research with drawing blood. Research was seen as the search for common health problems.

Contrastingly, some participants raised concerns, giving examples of what they considered to be poor research practices. For example, one participant mentioned that there were several projects that he had seen conducted by students from developed countries which he thought was not right.

Those people come to practice only. They did not come to give treatment. ……To me I think it is not good. Of course, they are in the developed countries, they can have modern equipment and different style of learning, but our knowledge, our experience won’t be lower than theirs. Because they all leave right after their practices the problems that are faced by the villagers later on are dealt with by us at the health centre.

(Villager, SSIs, Villages)

### Attitudes towards research

The main attitudes expressed towards research conducted in the community were trust of the researcher and their institution, someone to take responsibility for the research process and participation in it, the importance of feeding back findings and the provision of incentives. These are discussed in turn below.

#### Trust

From every question asked during the study, the theme of trust emerged and it included both trust of the organisation and trust of the individual researcher. Almost all of the participants said that recognition of the organisation and of the researcher’s good attitudes, such as being polite, respectful, confident, and appropriately dressed, enhanced trust. Regardless of the research topic, they would agree if they trust the researchers.

The researchers’ attitudes are important to gain trust from us. Showing the name of an established organisation that they are part of is important. Then the community members would know who they are and which organisation they are from.

(Mother, SSIs, Hospital)

It is also very important for participants to be able to clearly identify the researchers’ affiliation. For example, showing an introductory letter from their organisation and displaying the logo of the organisation are ways in which researchers can help enhance the trust between them and the participants.

If you come from the hospital you should wear a uniform with the hospital logo that we know……and also you should have some affiliated documents from the hospital. Then it will be very clear for us to know who you are.

(Parent, FGDs, Hospital)

However, institutional trust appeared to be more important for the participants recruited from AHC. They did not feel they needed evidence of a researcher’s affiliation to the hospital if the research was conducted in the hospital, however if researchers were to go out into the community they would need to clearly identify themselves.

In addition to their views about individual researchers and institutions, the participants often placed a great deal of importance on the role of the headman and the fact that before any research could be conducted in their village, the researchers must seek permission from the village headman.

A proper announcement from the village headman is needed, therefore it is important to let him know …If the villagers need more information about the study, they will ask the village headman. So if he does not know it would be problem. Villagers won’t trust you.

(Parent, SSIs, Hospital)

They argued that the village headman should be present with researchers while they conduct the study in order to make villagers feel confident to join the study.

I will not allow researchers to draw (blood) unless the village headman comes along with them, because I cannot be sure that permission was asked, because I cannot read what's in those document.

(Villagers, FGDs, Villages)

This finding contrasted with the village headman’s own account of their role, in that the village headmen said that their presence (or not) was up to the researchers, rather than being obligatory.

It would be based on the researchers’ requirements. If it is required then I will ask one of my team to go with them. Sometimes it can be me that goes or the vice village headman, but if they (researchers) are happy to go by themselves, then its fine, they can do it independently.

(Village headman, SSIs, Villages)

This difference suggests that views about responsibility and decision-making authority differed: and this was another theme emerging from the data.

#### Responsibility

Responsibility was one of the main themes in the study. Participants referred to a ‘group responsibility’, preferring to take action as a group rather than individually. For example, participants said they preferred researchers not to approach them individually (when initially explaining the study) but preferred to be approached and to participate as a group. They would accept individual participation (e.g. individual interviews), but only if other villagers within the group were also going to participate, and where individual participation isn’t strictly necessary they preferred group participation. This is because in a group, participants would feel less scared and more comfortable if they could see other people doing the same thing. Furthermore the responsibility for the decision to participate, and the consequences of that decision, would be shared amongst the group. One participant used the example of vaccination to explain this point.

If all villagers do it (vaccination) then I would love to join as well, but if they don’t get the injection then I won’t have my children get it also.

(Mother, SSIs, Hospital)

Participants also spoke of individuals in authority helping them in the decision to participate in research.

Within AHC participants said that if they were illiterate and their child was admitted in hospital, they would ask a nurse or doctor to help them decide what to do. However, if they were in the villages, they said that they needed the village headmen to fulfil this role. Participants from the villages also echoed this sentiment. Village authorities and village headmen were identified by participants as the key people responsible for giving permission to conduct any community activity, including research activities. Villagers said that village headman are the men in power so their agreement and support is vital.

Village headmen are considered to be the parents of the village. Most of the time they make decisions and give permission for every activity that happens in the village.

(Villager, FGDs, Hospital)

Participants placed great emphasis on proper approval processes being followed, particularly obtaining permission from the village headmen:

Good research is agreed by the village headman……Any good research has to go through the whole network of authorities.

(Parents, FGDs, Hospital)

Make sure it is agree by all the levels of authority and make sure that researchers have given enough information and obtained permission from the headman.

(Villager, SSIs, Villages)

These quotes demonstrate that participants found it very important to follow local permission processes. Participants who were not village headmen said that obtaining approval from the village headman prior to approaching individual villagers was required.

**Fig 3:**
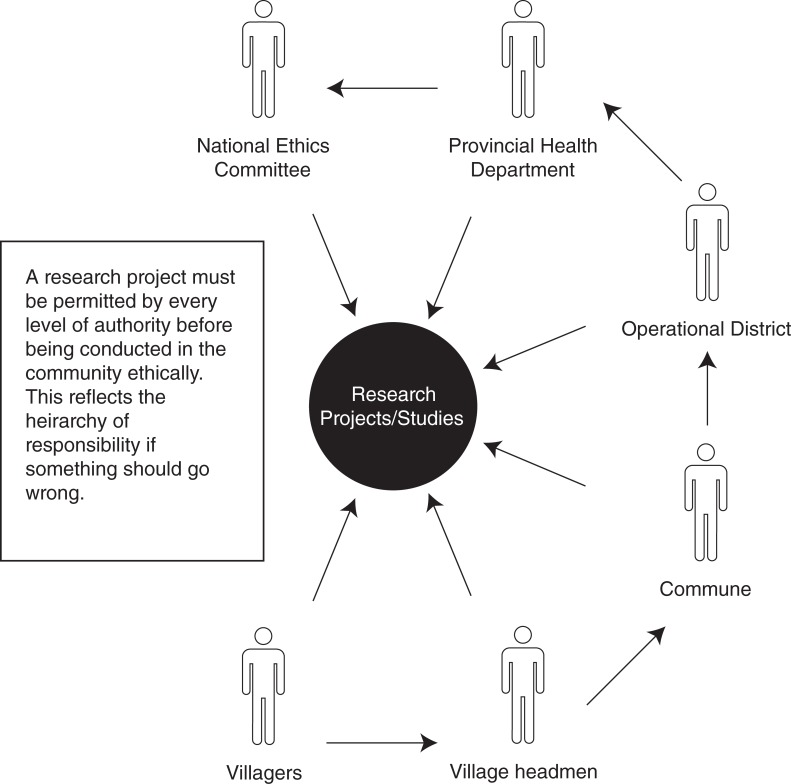
The network of approval needed before studies are allowed in the community.

Yet village headmen had a different point of view toward responsibilities. To them, responsibility is shared among a number of different people and involves the whole administrative system (as demonstrated in Fig 3).

First of all I need to be informed by the chief of the health centre, if it is related to healthcare, but if it is related to the administrative task I need to have permission from the chief of the commune. Otherwise I can’t make a decision.

(Village headman, SSIs, Villages)

Even though village headmen are the key people for villagers to rely on, village headmen only consider themselves as messengers to spread information to villagers after permission from higher levels is granted (Fig 3). Village headmen believed that their job is to be in the village to oversee and make processes go smoother.

Because the commune chief asked me, they have higher power than me. I am just a village headman so my job is to follow what they have agreed and then I would go to inform and collect the villagers. I also need to check with the commune chief because I need to make sure there is a person responsible for this.

(Village headman, SSIs, Villages)

Villagers also recognised that there is a chain of authority, which the village headmen are one part of. For them, the village headmen are the key people responsible for village affairs and their first point of contact, but they recognised that the village headmen also had levels of authority above them to whom they could refer.

We complain to the village headman, and the village headman will complain to the other levels above them.

(Villager, FGDs, Villages)

The perceptions and reality of responsibility within the community are complex, (as illustrated in Fig 3) although the idea of shared responsibility was common amongst responses from both the villagers and the village headmen, be it shared across a group of participants or shared across levels of authority.

### Attitudes towards consent

#### Methods of obtaining consent

Exploring attitudes towards the means of recording consent was an important aspect of this study. This study found that, in general, those asked were more comfortable providing verbal consent rather than providing a thumbprint or signature. If a physical form of consent had to be chosen then attitudes towards signing consent forms were similar between participants recruited from AHC and those recruited in the villages. As shown by the choices made on how participant chose to give consent for this study [S1 Table], participants preferred to sign rather than giving their thumbprint.

Furthermore, for some participants, providing a thumbprint or signature represents a situation in which they have no choice and feel their consent is mandated. This parent explains that:

When we don’t have choice and we have to agree with the doctor then we need to give thumbprint.

(Parents, FGDs, Hospital)

Some participants mentioned that the thumbprint and signature methods of gaining consent were both equally acceptable as they were certain on what they are agreeing to. However, most participants argued that giving a thumbprint is more serious in the consent process than giving a signature, because the former exposes their identity. A signature is considered to be less formal and with fewer negative repercussions as anyone can sign a form, but a thumbprint is unique to its owner. For this participant, the thumbprint is only given in situations where trust has been established:

I prefer giving my signature. I am worried that if I ever give my thumbprint they will make bad thing out of it. It's my thumbprint and my identity.…. But if it is something that I trust and if it is important, I want to give my thumbprint because it’s more reliable.

(Parent, FGDs, Hospital)

The perceptions of the community on providing their choice consent was very much dependent on how confidant they were with what they were agreeing to do.

#### The need for incentives

Some participants did not feel that receiving an incentive was important for any health activities. However, because previous organisations had frequently given incentives, participants had come to expect this as a normal part of any community activities including research activities [14].

It’s always nice to get that (gift) because it is like an incentive and thanks for participating in the study …Giving an incentive is not bribing and usually we get given it.

(Parent, FGDs, Hospital)

The above quote is important because it illustrates the whole process of how research should be conducted prior to the data collection process in the community from the community’s perspective.

## Discussion

This qualitative study was conducted in two locations in Siem Reap province, in North-Western Cambodia; Sotnikum health district and a children hospital [10]. Sotnikum health district comprises rural and peri-urban villages. Participants recruited from Sotnikum health district villages were likely to have had less exposure to health intervention and in turn healthcare research, in contrast to participants recruited from AHC who had accessed healthcare for their children and were more likely to have been involved or approached to be involved in healthcare research (research studies occur frequently at AHC). This difference in exposure to research and researchers may have impacted participants’ views. Sampling was the conducted pragmatically in both settings. Since AHC is an acute healthcare facility, parents and grandparents were recruited based on their availability at the hospital with respect to their caregiving duties. In the Sotnikum villages the community structure necessitated permission from the village headman first, only after which villagers could participate.

Participants recruited from the hospital and those from the villages of the Sotnikum heath district did not disagree significantly in their views about research. Whichever setting they came from, community members tended not to make a clear distinction between activities conducted for research and for general healthcare. A major contributing factor could have been that most rural health activities and healthcare research are performed at the same health facilities by a variety of different organisations. Often healthcare and healthcare research activities are both conducted by the same healthcare practitioners and at the same health centres or hospitals [15]. In practice, this is often to conserve resources and improve access to participants, but our analysis suggests that because of this researchers need to take extra care to ensure that participants understand clearly where the healthcare research activity differs from general healthcare [15–17].

One of the most important themes to emerge in this study is the extent to which participants rely on trust to make decisions about research participation. Views about trust took two different forms: those relating to personal trust and to institutional trust [4, 17]. Participants emphasised that researchers should be recognisable by the name of an established organisation and they must get permission from authorities at all levels (Fig 2). This is institutional trust which relates directly to institutions or companies, the government or social systems [4]. In addition to this, however, they should always behave respectfully and professionally in the community as individual people.

The importance of trust was also demonstrated when discussing consent. While consenting participants should never feel anxious after providing thumbprints or signatures [18]. Participants should be given the choice to document their consent with signature, thumbprint, verbal stated, or recorded verbal in order to help gain their trust. Trustworthiness should be maintained by balancing the power of all the main stakeholders to secure reliance and compliance. Therefore, it is vital for researchers to first build trust between them and participants, because once they trust researchers they will participate [4].

Another important theme related to the need for appropriate (as recognised by community members) authorisation. Researchers should have a good understanding of this before conducting research in any particular community and ensure that all parties are satisfied that proper approvals have been obtained. In addition to feeling confident that approval had been obtained properly, results from this study show that obtaining permission in the correct manner greatly enhances participants’ trust in the researchers. This is because, if approval has been obtained in the correct way, the village headman will have given his approval, in which case villagers can feel confident because the responsibility for their participation will also lie with the village headman.

Villagers directed responsibility to the village headman, stating that if the village headman approved then they would participate. Village headmen directed responsibility toward higher levels of authority, stating that if they got approval from them then they could authorise the villagers to participate. These differing attitudes of the villagers and village headmen toward where the responsibility lies for the decision to participate in research (and therefore of any negative consequences of this participation) could be explained by the phenomenon of ‘saving face’. In order to protect themselves from blame, and avoid embarrassment or punishment, and therefore to not ‘lose face’ villagers defer responsibility to the village headman who in turn defers responsibility to higher authorities [19]. When they get permission from the person above them, they feel more secure as they know they are not alone in making any mistakes [17]. This approach towards responsibility could explain the vital importance of following the required approval process in order for research activities to occur.

Interestingly, whilst it is often considered good practice to conduct research with individual participants as much as possible, this study found that participants felt more secure in a group environment. It was very important to them that the initial approach by the researcher and their subsequent decision to participate was made as a group. Participants would accept data collection to occur individually, but their participation in this was dependant on other group members also participating. It was clear that acting as a group made participants feel more secure. This may also relate to the concept of “saving face” as discussed earlier, as shared responsibility would prevent any one individual shouldering the blame for any untoward consequences. Awareness of the importance of the group will help researchers approach community members in the most appropriate way for them, helping to build trust and to conduct ethical research. It is important to be aware of this and to follow required processes to ensure that the all groups within the community feel confident in participating in research.

It can be seen from our findings that these key themes are all interlinked. Researchers must be aware of all these connected factors, so that community members can comfortably and confidently participate in research. There is often a gap in understanding between researchers and community members. This gap can be bridged by a good understanding of the factors that are important to community members, such as using clear terminology to explain the research activities, obtaining permission in the correct way from the correct authorities, approaching community members in the correct way, all of which ultimately lead to trust between community members and researchers. This trust is key in community members feeling secure and able to participate in research.

## Conclusion

Understanding community attitudes towards research by asking community members what their priorities are for research activities and what is acceptable to them is vital in order to bridge the gap between community members and researchers. These questions can find the right balance between researchers’ and community members’ goals and agenda and can help in maintaining better collaboration and accuracy of research outcomes [3]. If researchers are to build trust with communities, it is vital that they have a good understanding of the community structure and approvals process and that they make efforts to ensure that community members understand and know the organisation they are from. Mutual understanding between researchers and participants will enable high quality community-based research to be conducted.

## Supporting information

S1 Table Demographic information. (PDF)
