# Supplementary material for: If you come from a well-known organisation, I will trust you: Exploring and understanding the community’s attitudes towards healthcare research in Cambodia
Source: PLoS One. 2018 Apr 18;13(4):e0195251. doi: 10.1371/journal.pone.0195251 (PMC5905956; doi:10.1371/journal.pone.0195251)
Supplement: S1 Table — (PDF) [file pone.0195251.s001.pdf]

Table 1. Demographic information

	AHC n (%)	SOTNIKUM VILLAGES n (%)
**Number of participants in FGDs (4 groups) n=17**	9 (64.3)	8 (61.5)
**Number of participants SSIs n=10**	5 (35.7)	5 (35.7)
**Median age years (range)**	39 (23-54)	35 (17-65)
**Male n (%)**	6 (42.8)	7 (53.8)
**Known as**		
**Mother**	7 (50)	3 (23.2)
**Father**	6 (42.9)	0
**Villager (unmarried)**	0	5 (38.5)
**Grandmother**	1 (7.1)	1 (7.7)
**Healthcare worker**	0	1 (7.7)
**Village headman**	0	3 (22.4)
**Education level**		
**No education**	5 (35.7)	3 (23.1)
**Primary**	8 (56.9)	4 (30.8)
**Lower secondary**	1 (7.1)	0
**High school**	0	1 (7.7)
**Higher education**	0	5 (38.5)
**Choice of consent (more than one could be chosen)**		
**Thumbprint (n=14)**	7 (25.9)	7 (25.9)
**Signature (n=19)**	7 (25.9)	12 (44.4)
**Voice Recorded (n=5)**	5 (18.5)	0
**Have both self or their child participated in research before**	12 (85.7)	8 (61.5)
**Yes**	2 (14.3)	5 (38.5)
**No**		

Table 1 shows that there were equal numbers of participants from AHC and Sotnikum villages in the SSIs and FGDs. The median age and proportion of males from each location were also roughly equal. More mothers than fathers participated, but overall the gender balance was roughly equal among participants. The majority of participants had no education or only primary education. All participants that had higher levels of education were from Sotnikum villages. Regarding the choice of consent, participants indicated that both thumbprint and signature were acceptable, with most participants preferring to sign.
